# UBQLN2 Promotes the Production of Type I Interferon via the TBK1-IRF3 Pathway

**DOI:** 10.3390/cells9051205

**Published:** 2020-05-13

**Authors:** Tianhong Chen, Wenjuan Zhang, Bo Huang, Xuan Chen, Cao Huang

**Affiliations:** Department of Pathology, Anatomy & Cell Biology, Thomas Jefferson University, 1020 Locust Street, Philadelphia, PA 19107, USA; th.chen@siat.ac.cn (T.C.); Wenjuan.zhang@jefferson.edu (W.Z.); jason.huang777@yahoo.com (B.H.); cxchristina2018@gmail.com (X.C.)

**Keywords:** ALS, FTD, UBQLN2, TBK1, IFN1, IRF3

## Abstract

Mutations of Ubiquilin 2 (*UBQLN2*) or TANK-binding kinase 1 (*TBK1*) are associated with amyotrophic lateral sclerosis and frontotemporal degeneration (ALS/FTD). However, the mechanisms whereby *UBQLN2* or *TBK1* mutations lead to ALS and FTD remain unclear. Here, we explored the effect of UBQLN2 on TBK1 in HEK-293T cells or in CRISPR–Cas9-mediated IRF3 and IRF7 knockout (KO) cells. We found an interaction between TBK1 and UBQLN2, which was affected by ALS/FTD-linked mutations in *TBK1* or *UBQLN2*. Co-expression of UBQLN2 with TBK1 elevated the protein level of TBK1 as well as the phosphorylation of TBK1 and IRF3 in a UBQLN2 dose-dependent manner, and this phosphorylation was reduced by mutant UBQLN2. In addition, the cellular production of IFN1 and related pro-inflammatory cytokines was substantially elevated when UBQLN2 and TBK1 were co-expressed, which was also decreased by mutant UBQLN2. Functional assay revealed that mutant UBQLN2 significantly reduced the binding affinity of TBK1 for its partners, including IRF3, (SQSTM1)/p62 and optineurin (OPTN). Moreover, complete loss of IRF3 abolished the induction of IFN1 and related pro-inflammatory cytokines enhanced by UBQLN2 in HEK-293T cells, whereas no significant change in IRF7 knockout cells was observed. Thus, our findings suggest that UBQLN2 promotes IRF3 phosphorylation via TBK1, leading to enhanced IFN1 induction, and also imply that the dysregulated TBK1-IRF3 pathway may play a role in UBQLN2-related neurodegeneration.

## 1. Introduction

Mutations in the autophagy receptor Ubiquilin 2 (*UBQLN2*) have been associated with amyotrophic lateral sclerosis and frontotemporal degeneration (ALS/FTD) [1]. *UBQLN2* is an X-linked gene. The protein has an N-terminal ubiquitin-like domain (UBL), a C-terminal ubiquitin-associated domain (UBA), four heat-shock chaperonin-binding STI1 motifs, and a proline-rich disordered domain (PXX) [2]. Both UBL and UBA domains participate in interactions with proteasomes, and in autophagic protein degradation [3,4]. As a ubiquitin-like protein, it is known that most ALS/FTD-causing mutations in *UBQLN2* are within the PXX region, leading to the impairment of its proteasomal function [5,6]. Recent reports have also revealed that mutant UBQLN2 compromises autophagy in transgenic rodents [7,8,9], and stress granule formation are impaired by ALS/FTD-linked UBQLN2 mutations [10]. 

Genetic studies have also reported that TANK-binding kinase 1 (*TBK1*) mutations resulting in haploinsufficiency cause ALS and FTD [11,12,13]. There is no cure for ALS/FTD and the exact pathogenic mechanisms of these related disorders remain unclear. TBK1 is a ubiquitously-expressed 729 amino acid protein containing four functional domains: the serine/threonine (S/T) protein kinase domain (KD); the ubiquitin-like domain (UBL) and the coiled-coil domains 1 and 2 (CCD1/2) [14]. Mutations of *TBK1* that cause ALS/FTD occur throughout the coding region of *TBK1*, rather than clustering within any specific domain [15]. These ALS/FTD-linked mutations cause the accumulation of microtubule-associated protein 1 light chain 3 (LC3) and sequestosome 1 (SQSTM1)/p62, suggesting that malfunction of the autophagic pathway play a key role in the pathogenesis of neurodegeneration [7,8,9,16,17,18]. In mice, complete loss of TBK1 leads to embryonic death at approximately embryonic day 14.5 [19], whereas loss of one TBK1 allele has biphasic effects in mice that harbor the ALS-linked superoxide dismutase 1 (*SOD1*)-G93A mutation, accelerating the disease onset in the early stage but reducing microglial neuroinflammation and slowing disease progression at a later stage [19].

Reduced kinase activity is a major consequence of certain ALS/FTD-linked *TBK1* mutations [15]. TBK1 is a multi-functional protein kinase, but is best known as an innate immune kinase that mediates the activation of interferon regulatory factor 3/7 (IRF3/7) to induce the production of IFN1 (IFN-α and IFN-β) and related pro-inflammatory cytokines [20,21]. IRF3 promotes the production of primarily IFN-β [22], while IRF7 induces the production of both IFN-α and IFN-β [23]. As a protein kinase, the interactions between TBK1 and autophagy receptors have been well investigated. TBK1 was recently reported to phosphorylate and promote autophagy by interacting with the autophagy receptors OPTN [6,24] and SQSTM1/p62 [25,26], both of which were reported to be involved in the pathogenesis of ALS/FTD by impairing autophagy [27]. Two autophagic markers, LC3 and p62, are increased in TBK1-deficient mice, suggesting that TBK1 depletion impair autophagy [28]. However, little is known regarding the role of UBQLN2 in TBK1–IRF3 signaling. 

In the present study, we explored the effect of UBQLN2 on TBK1, and we found that over-expression of UBQLN2 increased TBK1 protein stability and TBK1 physically interacted with UBQLN2. Co-expressing UBQLN2 and TBK1 significantly enhanced the phosphorylation of both TBK1 and IRF3, leading to enhanced IFN1 production, whereas *UBQLN2* mutations impaired the phosphorylation of these proteins. Compared to wild-type UBQLN2, mutant UBQLN2 disrupted the interaction of TBK1 and its partner proteins, including IRF3, p62 and OPTN. In addition, the effects of UBQLN2 on production of IFN1 and related cytokines were abolished by the complete loss of IRF3 in HEK-293T cells but were not affected by the depletion of IRF7. All these findings suggest that UBQLN2 can promote IFN1 production via IRF3, and that dysregulation of this signaling pathway may play a critical role in UBQLN2-related diseases.

## 2. Materials and Methods

### 2.1. Plasmid Construction

Plasmids were constructed as previously described [29]. To increase the sensitivity of detection, human UBQLN2 cDNA were fused with the C-terminal 3x FLAG tag as well as the GFP tag at the N-terminus. Human TBK1 was synthesized and cloned into the pcDNA3 vector, which was used to express human TBK1 with a Myc tag at the C-terminus. All truncated UBQLN2 and TBK1 fragments were generated by PCR-based cloning, and all plasmids used in this study were sequenced to verify open reading frame sequence before transfection. Flag tagged IRF3, IRF7 and OPTN were purchased from GenScript (NJ, USA).

### 2.2. Cell Culture and Transfection

HEK-293T cells were obtained from American Type Culture Collection (ATCC, USA) and were cultured in Dulbecco’s Modified Eagle Medium supplemented with 10% fetal bovine serum (FBS) and antibiotics (ampicillin and streptomycin). For immunoprecipitation, cells were cultured in 6-cm dishes. At 70% confluence, cells were transfected with plasmids in the presence of FBS and absence of antibiotics, using lipofectamine-2000 according to the manufacturer’s instructions (Life Technologies, Grand Island, NY, USA). Cells were harvested at 24 h after transfection for RNA extraction, or at 48 h for immunoblotting.

### 2.3. CRISPR-Cas9–Mediated Knockout Cells

To generate IRF3 KO cells and IRF7 KO cells, HEK-293T cells were transfected with modified Cas9-vector (Addgene, MA, USA: #48138) harboring the IRF3 gRNA sequence (AAGGGATGCGGAAGCGCGTGCGG) or the IRF7 gRNA sequence (CTGGAAGCACTTCGCGCGCAAGG). Five hundred cells were seeded in 10-cm dishes at the next day of transfection and propagated for one week. Forty-eight single clones from single cells were picked for each sgRNA transfection. All picked colonies were propagated and were genotyped by immunoblotting with IRF3 or IRF7 antibody, and further confirmed by DNA Sanger sequencing.

### 2.4. Immunoprecipitation and Immunoblotting

Harvested cells were lysed in protein extraction buffer (50 mM Tris HCl, pH 7.4, with 150 mM NaCl, 1 mM EDTA, and 1% TRITON X-100) for 30 min on ice. Cell debris was removed by centrifugation at 16,000× *g* for 10 min. Protein concentrations of lysates were measured by Pierce BCA Protein Assay Kit. Five hundred micrograms total protein per sample was incubated with anti-FLAG-M2 Affinity Gel (Sigma) or anti-c-myc Affinity agarose gel (ThermoFisher Scientific, USA). Affinity resins were thoroughly washed with washing buffer (50 mM Tris HCl, 2.7 mM KCl, 137 mM NaCl, pH 7.4) at 4 °C to remove nonspecifically bound proteins. Bound proteins were eluted with sodium dodecyl sulfate sample buffer (100 mM Tris HCl, 12.5 mM EDTA and 2% sodium dodecyl sulfate (SDS), pH 7.4) and boiled for 5 min followed by immunoblotting. Equal volumes for each sample were resolved by sodium dodecyl sulfate–polyacrylamide gel electrophoresis (SDS-PAGE) for immunoblotting, as described previously [8]. Resolved proteins were transferred to nitrocellulose membranes for detection with the following primary antibodies: rabbit anti-TBK1 (Abcam, USA, 1:1000), rabbit anti-p-TBK1^s172^ (Abcam, USA, 1:1000), rabbit anti-IRF3 (Abcam, 1:1000), rabbit anti-p-IRF3^s386^ (Abcam, 1:1000), rabbit anti-IRF7 (Cell Signaling Technology, USA, 1:1000), mouse anti-UBQLN2 (Abnova: 1:1000), rabbit anti-myc (GenScript, 1:1000), mouse anti-Flag (Sigma, 1:1000) or rabbit anti-Flag (GenScript, 1:1000), rabbit anti-p62 (Proteintech, USA, 1:2000), rabbit anti-OPTN (Proteintech, 1:1000), mouse anti-GFP (Proteintech, 1:5000), and mouse anti-GAPDH (Proteintech, 1:5000). 

### 2.5. Glutathione S-Transferase (GST) Pull-Down Assay

To assess in vitro binding interactions, recombinant His-tagged UBQLN2 (His-UBQLN2: Abcam) and GST-tagged TBK1 (GST-TBK1: Thermo Fisher) were combined in 800 μL of 2% Chaps buffer (20 mM Tris pH 8.0, 150 mM NaCl, 2 mM MgCl_2_, 10% glycerol, 2.0% Chaps). Then, 70 μL of equilibrated glutathione-Sepharose 4B (GE Healthcare, USA) was added, and the mixture was incubated for 2 h with rotation. The mixture was centrifuged at 5000 rpm for 5 min, and the supernatant was discarded. The beads were washed four times with 1× PBS containing 2% Tween 20 and 200 mM KCL, once with 1× PBS, then resuspended in SDS sample buffer to give 100 μL. Samples were heated for 5 min at 100 °C, and analyzed by SDS–PAGE followed by immunoblotting.

### 2.6. RNA Extraction and Quantitative PCR Analysis

HEK-293T cells were transfected with myc-tagged TBK1 and Flag-tagged UBQLN2. Total RNA was extracted with Trizol reagent (Thermo Fisher Scientific) 24 h after transfection. Total RNA (1 μg) was used for reverse transcription with ProtoScript First Strand cDNA Synthesis Kit (New England Biolabs, USA). Amplifications of qPCR analysis were performed with SYBR green in an Applied Biosystems Real-Time PCR Instrument (Thermo Fisher Scientific) using the following primers: hIFN-β-F, GACTTACAGGTTACCTCCGAAA, hIFN-β-R, CATATGCAGTACATTAGCCAT; hIFN-α-F, TGACAGAGAAGAAATACAGCC, hIFN-α-R, ATTGTTTTCATGTTGGACCAG; hIL1-F, TACAGCTGGAGAGTGTAGATCCC, hIL1-R, GCAGACTCAAATTCCAGCTTGTT; hIL6-F, TGTGCAGATGAGTACAAAAGTCCT, hIL6-R, ATGTCCTGCAGCCACTGGTTC; hIL8-F, GCCAACACAGAAATTATTGTAAAGC, hIL8-R, CTGGCATCTTCACTGATTCTTG; hISG54-F, CTTCCCAGTCTATCATCAACCTT, hISG54-R, CCGTCGCTTCTAGCTATGTATCT; hISG56-F, TCATCAGGTCAAGGATAGTC, hISG56-R, CCACACTGTATTTGGTGTCTAGG. Target mRNA levels were normalized to GAPDH mRNA levels as an internal control.

### 2.7. Dual-Luciferase Reporter Assay

A Dual-Luciferase^®^ Reporter Assay System was purchased from Promega and used as per the manufacturer’s instructions. HEK-293T cells were transfected with an IFN-β (Firefly luciferase) and a Renilla-TK reporter (Addgene), along with wild-type TBK1 and UBQLN2 or the P497H or P506T mutant forms. Cells were harvested 24 h after transfection and lysed following the manufacturer’s instructions. Luciferase activity in the lysates were measured by Promega™ GloMax^®^ Plate Reader. Data for IFN-β were normalized to the Renilla luciferase internal control.

### 2.8. Statistical Analysis

All data shown are representative and all experiments were repeated at least three times independently. Statistical analysis was carried out using one-way ANOVA or unpaired *t* tests. *p* < 0.05 was considered statistically significant. 

## 3. Results

### 3.1. Interactions between TBK1 and UBQLN2

**Figure 1:**
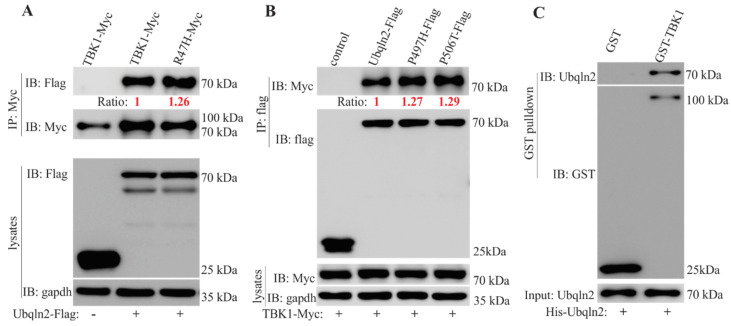
TANK-binding kinase 1 (TBK1) interacts with Ubiquilin 2 (UBQLN2). (**A**,**B**) Immunoprecipitation (IP) revealed that TBK1 interacted with UBQLN2 in HEK-293T cells when co-transfected with plasmids (Myc-tagged TBK1 and Flag-tagged UBQLN2) using Lipofectamine 2000. The transfected cells were harvested 48 h after transfection. The ratios in panel A were calculated based on the pull-downs of myc-tagged baits, and in panel B, these ratios were calculated according to the pull-down of the Myc or Flag-tagged bait protein Control: GFP tagged with Flag. (**C**) Glutathione-S-transferase (GST) pull-down assay revealed that UBQLN2 bound to TBK1 in vitro. Recombinant UBQLN2 protein was incubated with GST or GST-TBK1 protein for 2 h, and GST was then pulled down with glutathione-agarose beads. Proteins in the precipitates were detected by immunoblot with UBQLN2 and GST antibodies.

TBK1 phosphorylates and interacts with three autophagy receptors: OPTN [6,24], SQSTM1/p62 [25,26], and NDP52 [30]. Mutations in both *p62* and *OPTN* have been reported to cause ALS/FTD [27], as do mutations in another autophagy receptor protein, UBQLN2 [1]. To our knowledge, the interplay of TBK1 and UBQLN2 has not been reported. To examine this, we used immunoprecipitation (IP) to detect interaction between TBK1 and UBQLN2 in HEK-293T cells. TBK1 and UBQLN2 tagged at the C-terminus with myc and Flag, respectively, were co-expressed in HEK-293T cells, and immunoaffinity resins were used to isolate the respective complexes. We noted that UBQLN2 was consistently immunoprecipitated with TBK1-Myc, and vice versa (Figure 1A,B). Intriguingly, we found that pathogenic mutations in either *TBK1* (R47H substitution) or *UBQLN2* (P497H and P506T substitution) enhanced their binding to one another (Figure 1A,B). Additionally, we co-transfected TBK-myc and GFP-UBQLN2 and IP with anti-c-myc resins, and revealed that the two proteins were precipitated (Figure S1). These results suggest that there is an interaction between UBQLN2 and TBK1. To further verify an interaction between TBK1 and UBQLN2, we conducted an in vitro glutathione S-transferase (GST) pull-down assay. As shown in Figure 1C, His-UBQLN2 was effectively pulled down with GST-TBK1, thereby suggesting a direct interaction between UBQLN2 and TBK1.

**Figure 2:**
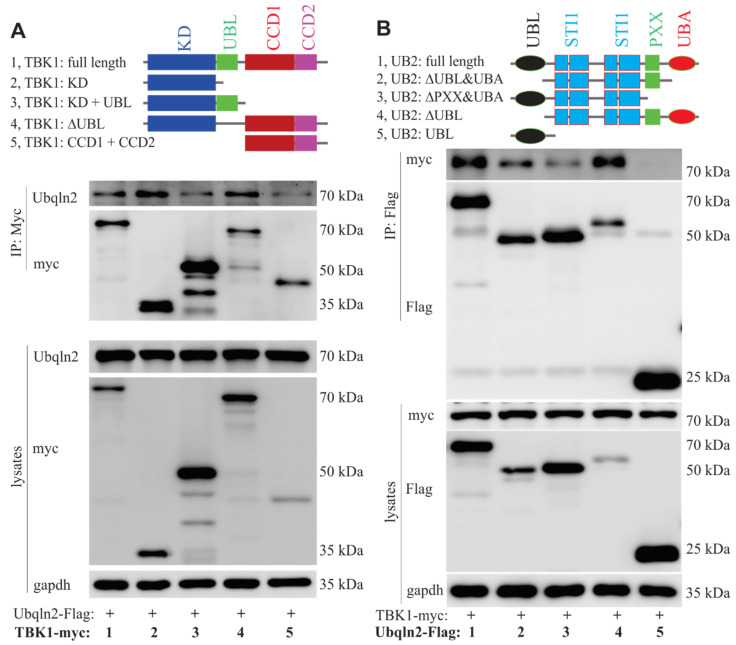
Binding domains of TBK1 and UBQLN2. (**A**) Use of IP and immunoblotting to define the TBK1 domain that binds to UBQLN2. HEK-293T cells were co-transfected with full length or mutant TBK1 and UBQLN2-3xFlag. Cells were harvested 48 h after transfection. KD, the serine/threonine (S/T) protein kinase domain; UBL, the ubiquitin-like domain; CCD1/2, the coiled-coil domains 1 and 2; ∆UBL, TBK1 lacking the UBL domain. (**B**) The region of UBQLN2 binding to TBK1 was similarly defined. HEK-293T cells were transfected with TBK1-myc, along with either full-length UBQLN2 or truncated UBQLN2. UB2, UBQLN2; UBL, the ubiquitin-like domain; STI1, heat-shock chaperonin-binding motifs; PXX, the proline-rich disordered domain; UBA, ubiquitin-associated domain; ∆UBL, UBQLN2 lacking the UBL domain; ∆UBL&UBA, UBQLN2 lacking both UBL and UBA; ∆PXX&UBA, UBQLN2 lacking both PXX and UBA.

To identify the domains in TBK1 and UBQLN2 that are responsible for the interaction, we created TBK1 truncated mutants (harboring a deletion of KD, UBL, or CDD1/2 domain) with the C-terminal myc-tag and UBQLN2 truncated mutants (harboring a deletion of UBL, STI, PXX or UBA domain) with the C-terminal 3x FLAG tag. The immunoprecipitation of TBK1-myc or its truncated mutants with UBQLN2-Flag showed that TBK1 bound to UBQLN2 mainly via its protein kinase and CCD1/2 domains (Figure 2A). As shown in Figure 2B, TBK1 was not pulled down with truncated UBQLN2 harboring only UBL domain and removal of UBA domain substantially decreased the binding affinity of UBQLN2 for TBK1, suggesting that the UBA domain of UBQLN2 is critical for its interaction with TBK1 (Figure 2B).

### 3.2. Over-Expression of UBQLN2 Positively Promotes TBK1 Phosphorylation

**Figure 3:**
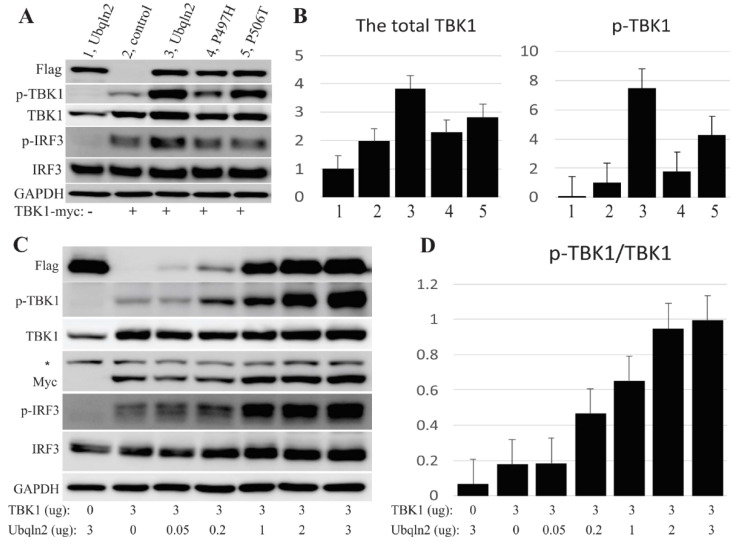
UBQLN2 promotes TBK1 phosphorylation. (**A**) Immunoblot analysis revealed the expression levels of TBK1 and IRF3 when co-expressed with UBQLN2. The empty vector was co-transfected as the control. Each lane was loaded with 10 µg total protein, and GAPDH was used as the loading control. Human UBQLN2 and TBK1 were tagged with Flag or Myc and co-transfected into HEK-293T cells. (**B**) Graphs showing the relative levels of TBK1 based on GAPDH and the relative levels of p-TBK1 based on TBK1, calculated from the panel (**A**). (**C**) HEK-293T cells that were seeded into 10-cm dishes were co-transfected with TBK1 and varying amounts of UBQLN2 (0, 0.05, 0.2, 1, 2, 3 µg). Cells were harvested 48 h after transfection. Immunoblot reveals the levels of p-TBK1, TBK1, p-IRF3 and IRF3, in response to increasing expression of UBQLN2. An asterisk indicates non-specific bands detected by anti-myc. (**D**) Ratios of p-TBK1 to total TBK1, calculated from Panel C. Note: the total TBK1: TBK1; the phosphorylated TBK1: p-TBK1; the total IRF3: IRF3; the phosphorylated IRF3: p-IRF3.

One of the best-known functions of TBK1 is mediating IFN1 signaling via the phosphorylation of IRF3/7 [20,21,31]. To determine the role of UBQLN2 in TBK1-IRF3, we co-transfected HEK-293T cells with TBK1 and UBQLN2. Immunoblotting showed that both phosphorylated TBK1 (p-TBK1) and total TBK1 were significantly elevated, and IRF3 phosphorylation was increased as well (Figure 3), indicating that UBQLN2 may stabilize TBK1, thereby enhancing IRF3 phosphorylation. Moreover, UBQLN2 mutants harboring ALS-FTD-linked mutations were substantially less effective than wild-type UBQLN2 in promoting TBK1 phosphorylation (Figure 3). As previously reported [11,13,15], the R47H mutation in *TBK1* lead to impaired phosphorylation of TBK1 and IRF3, which is consistent with our results (Figure S2).

To further investigate the effect of UBQLN2 on TBK1 protein, we co-transfected HEK-293T cells with TBK1 and varying amount of UBQLN2. As shown in Figure 3C,D, increases in the levels of TBK1 and IRF3 were correlated with changes in UBQLN2 expression. Moreover, increasing amounts of UBQLN2 led to progressively greater phosphorylation of TBK1 and IRF3. The UBQLN2 dose-dependence of TBK1 phosphorylation suggested to us that UBQLN2 might stabilize TBK1 protein in cells. Therefore, we co-transfected cells with TBK1 and either UBQLN2 expression vector or an empty vector, and added cycloheximide 24 h later. We then used immunoblotting to determine TBK1 levels at varying times after cycloheximide addition (Figure S3). The results of immunoblotting show that UBQLN2 co-expression slowed the degradation of the TBK1.

### 3.3. UBQLN2 Promotes IFN1 Production

**Figure 4:**
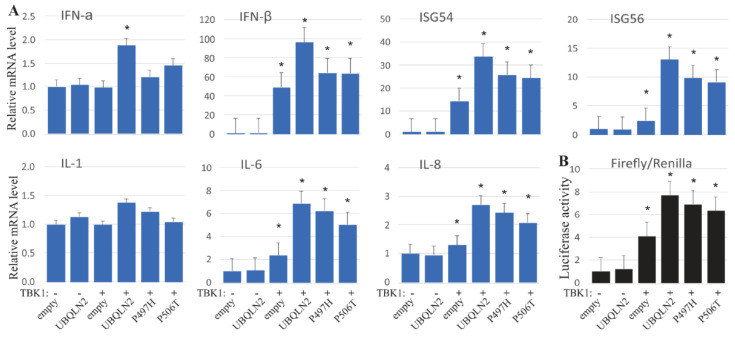
UBQLN2 promotes type 1 IFN production. (**A**) Quantitative PCR analysis revealed the relative levels of mRNA for IFN1 and related cytokines. HEK-293T cells. were transfected with Myc-tagged TBK1 and Flag-tagged wild-type or mutant UBQLN2. Total RNA was extracted with Trizol reagent 24 h after transfection. (**B**) Luciferase reporter assay showing the IFN-β activities. HEK-293T cells were transfected with an IFN-β (luciferase) and a Renilla-TK reporter from Addgene, along with wild-type TBK1 and UBQLN2 or their mutant forms: P497H or P506T, respectively. Assays were performed 24 h after co-transfection of TBK1 and UBQLN2. The data were calculated based on the empty vector, and are reported as the mean ± standard deviation (n = 3). * *p* < 0.05.

Activated TBK1 and IkB kinase-ε can phosphorylate IRF3, promoting the production of IFN1 and related pro-inflammatory cytokines [31,32,33]. We next tested the effect of UBQLN2 co-expression on IFN1 and related cytokines in HEK-293T cells expressing TBK1. Quantitative PCR showed that mRNA level of IFN-β, IL-6, IL-8 and the interferon induced genes (ISGs) ISG54 and ISG56 were substantially increased by co-expression of UBQLN2, whereas the levels of IFN-α and IL-1 were slightly elevated (Figure 4A). And the ALS/FTD-linked *UBQLN2* mutations (P497H and P506T) diminished these productions (Figure 4A). In a second approach, the expression of IFN-β was evaluated using a luciferase reporter assay. The reporter assay confirmed that UBQLN2 promoted IFN-β production, and that the pathogenic *UBQLN2* mutations (P497H and P506T) impaired its ability to enhance TBK1-dependent IFN-β production (Figure 4B). The results suggest that ALS/FTD mutations in *UBQLN2* may undermine its role in TBK1-IRF3 signaling.

Similar to UBQLN2, another ubiquilin family member, ubiquilin 1 (*UBQLN1*), which also harbors the N-terminal UBL and C-terminal UBA domains as well as the STI motif [34]. To examine whether UBQLN1 has a similar effect on TBK1, we co-transfected UBQLN1 and TBK1 into HEK-293T cells. Compared to UBQLN2, the results of immunoblots show that no enhanced phosphorylation of TBK1 and IRF3 was observed when co-expressed with UBQLN1 (Figure S4), and we did not detect any significant alterations in IFN production and related pro-inflammatory cytokines (data not shown). Immunoprecipitation showed that the myc-tagged TBK1 did not precipitate with Flag-tagged UBQLN1 (Figure S4), suggesting that no interaction exist between TBK1 and UBQLN1, and increased UBQLN1 expression does not promote the phosphorylation of TBK1 and IRF3 as well. 

### 3.4. Mutant UBQLN2 Impair the Interaction between TBK1 and its Partners

**Figure 5:**
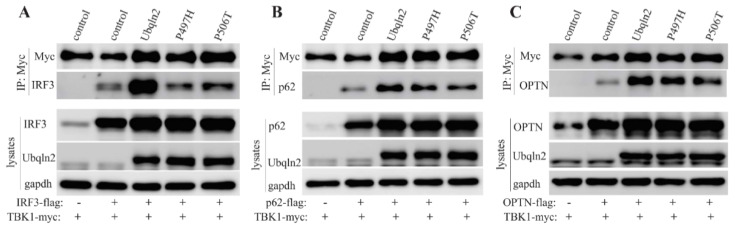
Mutant UBQLN2 impairs TBK1 interactions with partner proteins. HEK-293T cells were co-transfected with TBK1-myc and IRF3-flag or p62-flag or OPTN-flag as well as wild-type or mutant UBQLN2-3xFlag expression vector or empty vector. Harvested cells 48 h after transfection. IP with anti-c-myc resins (Myc-IP) followed by immunoblotting and probed with indicated antibodies. (**A**) Myc-IP to pull down IRF3. (**B**) Myc-IP to pull down p62. (**C**) Myc-IP to pull down OPTN.

TBK1 phosphorylates and also interacts with p62 and OPTN [6,25], as well as IRF3. To detect the effect of UBQLN2 on the interactions of TBK1 and its partners, we co-transfected UBQLN2 and TBK1 along with p62 or IRF3 or OPTN into HEK-293T cells. Myc IP showed that the co-expression of UBQLN2 enhanced the TBK1 binding affinity to its partners including IRF3 (Figure 5A), P62 (Figure 5B) and OPTN (Figure 5C). Compared to wild-type UBQLN2, ALS/FTD-linked UBQLN2 mutations significantly decreased the binding affinity of TBK1 for its partners (Figure 5). These results indicate that mutant UBQLN2 may impair the interactions of TBK1 with its partners, leading to the functional impairment of TBK1.

### 3.5. UBQLN2 Promotes IFN1 Induction via IRF3

**Figure 6:**
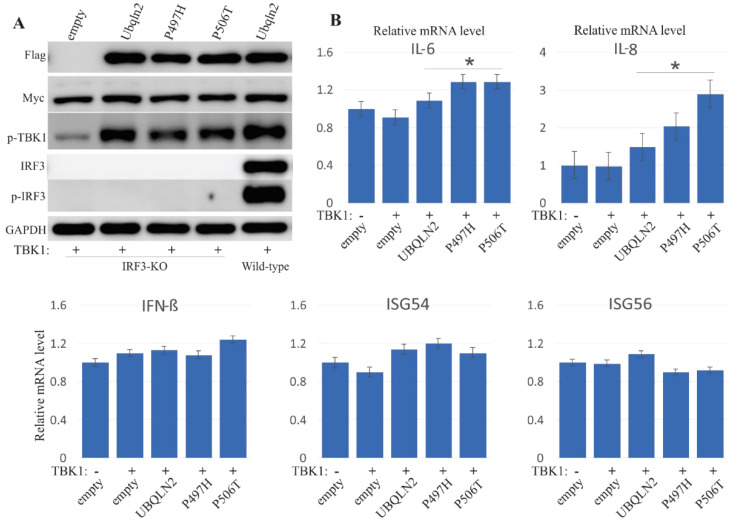
Loss of IRF3 abolishes the effect of UBQLN2 on IFN1 production. CRISPR–Cas9-mediated IRF3 knockout (KO) HEK-293T cells were transfected with Myc-tagged TBK1 and Flag-tagged wild-type or mutant UBQLN2. Harvested cells 24 h after transfection. (**A**) Immunoblot analysis revealed the expression levels of TBK1 when co-expressed UBQLN2 with TBK1 in IRF3-KO cells. (**B**) Quantitative PCR analysis revealed the relative levels of mRNA for IFN-β and related cytokines in IRF3-KO cells. The data are reported as the mean ± standard deviation (n = 3). * *p* < 0.05.

TBK1 phosphorylates IRF3 and IRF7, leading to IFN1 induction [35]. To examine whether UBQLN2 enhances the IFN1 production via IRF3 or IRF7, we generated CRISPR-Cas9–mediated IRF3 and IRF7 knockout (KO) cells (Figure 6A, Figure S5). In IRF3-KO cells, TBK1 phosphorylation was enhanced when co-expressed with UBQLN2 (Figure 6), which is consistent with previous results (Figure 3, Figures S2 and S4), whereas the mRNA of IFN-β, IL-6, ISG54 and ISG56 were not induced by TBK1 over-expression or co-expression of TBK1 and UBQLN2 in IRF3-KO cells, indicating that enhanced induction of IFN1 and related cytokines by UBQLN2 co-expression is dependent on IRF3. However, both immunoblots and qPCR showed similar results to those observed in wild-type cells when co-expressed TBK1 with UBQLN2 into IRF7-KO cells (Figure S5). All these results imply that UBQLN2 promotes the IFN1 inductions mainly via IRF3, rather than IRF7.

## 4. Discussion

Mutations in the ubiquitin binding protein *UBQLN2* impair protein degradation via autophagy and the ubiquitin-proteasome pathway [36,37,38,39], and accumulating evidence shows that this contributes to the development of ALS/FTD [1,9,40]. The functional impairment of TBK1 causes ALS/FTD [11,13,15], and ALS/FTD-causing mutations in *TBK1* that lead to TBK1 haploinsufficiency cause dysregulation of the TBK1-IRF3 pathway [11,12]. Here, we reported that mutant UBQLN2 also result in the impaired TBK1-IRF3 signaling. 

Two autophagy receptors, *p62* [41] and *OPTN* [42], have been reported to cause ALS and to interact with TBK1 [6,25]. Using IP and GST pull-down assays, we found that UBQLN2, another autophagy receptor, also interacted with TBK1. In addition, ALS/FTD-linked mutations, either in *UBQLN2* (P497H and P506T) or in *TBK1* (R47H), increased their mutual binding affinity. Similarly, ALS/FTD-linked mutations in *TBK1* disrupted its interactions with several autophagy receptors including p62, OPTN, and NDP52, leading to impaired autophagy [43]. Likewise, ALS/FTD-linked *UBQLN2* mutations cause impairment of autophagy [8,9].

TBK1 binds to adaptor proteins, such as OPTN, NAP1, and TANK, via its C-terminal region [44,45], which is consistent with our finding that TBK1 interacted with UBQLN2 primarily via the TBK1 coiled-coil domain as well as the protein kinase domain. The majority of UBQLN2 mutations that cause ALS/FTD are located within the PXX domain, which is important for interactions with its protein partners [1], and this is in line with our observation of stronger binding to TBK1 via the C-terminal region of UBQLN2. In future studies, more ALS/FTD-linked mutations should be explored in order to understand the functional roles of this interaction.

TBK1 is activated by its phosphorylation at Ser 172 [46]. In HEK-293T cells over-expressing TBK1, we observed both TBK1 autophosphorylation and phosphorylation of IRF3 by TBK1, identical to previous reports [31,32,33]. Co-expression of UBQLN2 with TBK1 enhanced the protein level of TBK1 as well as the phosphorylation of both TBK1 and IRF3, and this phosphorylation was reduced by ALS/FTD-linked mutations in *UBQLN2*, and no altered TBK1 and IRF3 phosphorylation was exhibited in cells that co-expressed with UBQLN1 with TBK1. This disparity may result from the unique PXX domain of UBQLN2 and it distinguishes UBQLN2 from other ubiquilin proteins. Additionally, we found that the phosphorylation of TBK1 and IRF3 was positively correlated with the level of UBQLN2 expression. Moreover, comparing rates of protein turnover in the presence of cycloheximide showed that UBQLN2 promoted TBK1 protein stability. We previously reported that both mutant human UBQLN2 and wild-type human/rat UBQLN2 form protein inclusions including p62 and Rpt1 [9,47], implying that the over-expression of UBQLN2 may cause the dysregulation of protein homeostasis, leading to toxicity. Proteasome impairment by mutant UBQLN2 was identified [1], while the ALS/FTD-linked mutations have been shown to cause defective proteasome delivery and to slow the degradation of UBQLN2 protein [40]. Collectively, these findings suggest the possibility that UBQLN2 over-expression may promote TBK1 expression by interfering with its degradation, and that proteasome function is impeded even more by mutant UBQLN2 than by wild-type form.

Phosphorylation of IRF3 by activated TBK1 is known to promote the production of IFNs and pro-inflammatory cytokines [31,32,33], and we obtained identical results in our study. Consistent with the effect of UBQLN2 on TBK1, in cells co-transfected with TBK1 and UBQLN2 we detected dramatically elevated expression of IFN-β and pro-inflammatory cytokines, as well as ISG54 and ISG56. The expression of IFN-β was more strongly affected than IFN-α. Lower cytokine expression was observed with pathogenic UBQLN2 than with wild-type UBQLN2. A luciferase reporter assay for IFN-β confirmed qPCR results indicating that UBQLN2 promoted IFN1 production, whereas the pathogenic UBQLN2 mutations reduced it. These findings indicate that UBQLN2 may play a role in regulating the TBK1-IRF3 pathway. Similarly, another autophagy receptor, OPTN, also positively promotes the production of TBK1 and IFN-β, which is impaired by ALS-linked mutant OPTN [48]. In SOD1-G93A mice, the interferon pathway is activated [49], whereas the ALS-linked TBK1 mutation decreases the interferon response and accelerates the disease onset [50]. All these findings suggest that impaired TBK1 proteins lead to the reduction in interferon production, and further indicate that dysregulation of immune response may involve in ALS.

ALS/FTD-linked mutations in *TBK1* lead to its functional impairment as well as disrupted interaction of TBK1 with its partners [15,51]. Here, we observed that the interaction between TBK1 and its several substrates, including IRF3, p62 and OPTN, were greatly disrupted by UBQLN2 mutations, implying that impaired TBK1 may contribute a role in the pathogenesis of UBQLN2-related ALS/FTD. In the future studies, genetic screening is still helpful to examine whether concurrent mutations of TBK1 and UBQLN2 are existed and whether impaired TBK1 exists in patients. To further understand how UBQLN2 mutations affect TBK1, studies on UBQLN2 animal models are applicable, and mutant UBQLN2 knock-in models would be valuable. As complete loss of TBK1 led to embryonic death in mice [19] and neuron specific deletion of TBK1 causes impaired autophagy [52], indicating that TBK1 is essential to mice. To better understand how pathogenic TBK1 mutations cause ALS, animal models harboring *TBK1*-linked ALS/FTD mutations including transgenic and knock-in animals are strongly recommended. Compound knock-in animal models harboring both *UBQLN2* and *TBK1* mutations would be ideal models to study the synergistic effects of concurrent *TBK1* and *UBQLN2* mutations. 

As reported, IRF3 promotes the production of primarily IFN-β [22], while IRF7 induces the production of both IFN-α and IFN-β [23]. In our current study, IFN-β production was abolished by the complete loss of IRF3, but no significant alteration in IRF7-KO cells was observed, suggesting that UBQLN2 promotes IFN1 production through IRF3 instead of IRF7. Our results as a whole indicate that mutant UBQLN2 cause functional impairment of TBK1. 

## 5. Conclusions

Taken together, we demonstrated that UBQLN2, rather than UBQLN1, promoted IRF3 phosphorylation via enhanced TBK1 phosphorylation, leading to the enhanced production of IFN1. *UBQLN2* with ALS-FTD-linked mutations caused functional impairment of TBK1 and reducing production of IFN1. Our new findings thus suggest a potential role of UBQLN2 in regulating IFN1 production.

## Supplementary Materials

The following are available online at https://www.mdpi.com/2073-4409/9/5/1205/s1, Figure S1: TBK1 precipitates UBQLN2. Figure S2: UBQLN2 has no effect on mutant TBK1. Figure S3: UBQLN2 increases TBK1 protein stability. Figure S4: Increased expression of Ubiquilin 1 does not affect TBK1 phosphorylation. Figure S5: Loss of IRF7 has no effect on IFN1 production enhanced by UBQLN2.

## References

[1] Deng H.-X., Chen W., Hong S.-T., Boycott K.M., Gorrie G.H., Siddique N., Yang Y., Fecto F., Shi Y., Zhai H. (2011). Mutations in UBQLN2 cause dominant X-linked juvenile and adult-onset ALS and ALS/dementia. Nature.

[2] Kleijnen M.F., Shih A.H., Zhou P., Kumar S., Soccio R.E., Kedersha N.L., Gill G., Howley P. (2000). The hPLIC proteins may provide a link between the ubiquitination machinery and the proteasome. Mol. Cell.

[3] Walters K.J., Kleijnen M.F., Goh A.M., Wagner G., Howley P. (2002). Structural studies of the interaction between ubiquitin family proteins and proteasome subunit S5a. Biochemistry.

[4] N’Diaye E.-N., Kajihara K.K., Hsieh I., Morisaki H., Debnath J., Brown E.J. (2009). PLIC proteins or ubiquilins regulate autophagy-dependent cell survival during nutrient starvation. EMBO Rep..

[5] Osaka M., Ito D., Suzuki N. (2016). Disturbance of proteasomal and autophagic protein degradation pathways by amyotrophic lateral sclerosis-linked mutations in ubiquilin 2. Biochem. Biophys. Res. Commun..

[6] Wild P., Farhan H., McEwan D.G., Wagner S., Rogov V.V., Brady N.R., Richter B., Korac J., Waidmann O., Choudhary C. (2011). Phosphorylation of the autophagy receptor optineurin restricts Salmonella growth. Science.

[7] Gorrie G.H., Fecto F., Radzicki D., Weiss C., Shi Y., Dong H., Zhai H., Fu R., Liu E., Li S. (2014). Dendritic spinopathy in transgenic mice expressing ALS/dementia-linked mutant UBQLN2. Proc. Natl. Acad. Sci. USA.

[8] Chen T., Huang B., Shi X., Gao L., Huang C. (2018). Mutant UBQLN2(P497H) in motor neurons leads to ALS-like phenotypes and defective autophagy in rats. Acta Neuropathol. Commun..

[9] Wu Q., Liu M., Huang C., Liu X., Huang B., Li N., Zhou H., Xia X.G. (2015). Pathogenic Ubqln2 gains toxic properties to induce neuron death. Acta Neuropathol..

[10] Alexander E., Niaki A.G., Zhang T., Sarkar J., Liu Y., Nirujogi R.S., Pandey A., Myong S., Wang J. (2018). Ubiquilin 2 modulates ALS/FTD-linked FUS-RNA complex dynamics and stress granule formation. Proc. Natl. Acad. Sci. USA.

[11] Freischmidt A., Wieland T., Richter B., Ruf W., Schaeffer V., Muller K., Marroquin N., Nordin F., Hübers A., Weydt P. (2015). Haploinsufficiency of TBK1 causes familial ALS and fronto-temporal dementia. Nat. Neurosci..

[12] Cirulli E.T., Lasseigne B.N., Petrovski S., Sapp P.C., Dion P.A., Leblond C.S., Couthouis J., Lu Y.-F., Wang Q., Krueger B.J. (2015). Exome sequencing in amyotrophic lateral sclerosis identifies risk genes and pathways. Science.

[13] Gijselinck I., Van Mossevelde S., Van Der Zee J., Sieben A., Philtjens S., Heeman B., Engelborghs S., Vandenbulcke M., De Baets G., Bäumer V. (2015). Loss of TBK1 is a frequent cause of frontotemporal dementia in a Belgian cohort. Neurology.

[14] Pomerantz J.L., Baltimore D. (1999). NF-kappaB activation by a signaling complex containing TRAF2, TANK and TBK1, a novel IKK-related kinase. EMBO J..

[15] Ye J., Cheung J., Gerbino V., Ahlsén G., Zimanyi C., Hirsh D., Maniatis T. (2019). Effects of ALS-associated TANK binding kinase 1 mutations on protein-protein interactions and kinase activity. Proc. Natl. Acad. Sci. USA.

[16] Oakes J.A., Davies M.C., Collins M.O. (2017). TBK1: A new player in ALS linking autophagy and neuroinflammation. Mol. Brain.

[17] Pilli M., Arko-Mensah J., Ponpuak M., Roberts E., Master S., Mandell M.A., Dupont N., Ornatowski W., Jiang S., Bradfute S.B. (2012). TBK-1 promotes autophagy-mediated antimicrobial defense by controlling autophagosome maturation. Immunity.

[18] Le N.T.T., Chang L., Kovlyagina I., Georgiou P., Safren N., Braunstein K.E., Kvarta M., Van Dyke A.M., LeGates T.A., Philips T. (2016). Motor neuron disease, TDP-43 pathology, and memory deficits in mice expressing ALS-FTD-linked UBQLN2 mutations. Proc. Natl. Acad. Sci. USA.

[19] Bonnard M., Mirtsos C., Suzuki S., Graham K., Huang J., Ng M., Itie A., Wakeham A., Shahinian A., Henzel W. (2000). Deficiency of T2K leads to apoptotic liver degeneration and impaired NF-kappaB-dependent gene transcription. EMBO J..

[20] Ishikawa H., Barber G.N. (2008). STING is an endoplasmic reticulum adaptor that facilitates innate immune signalling. Nature.

[21] Chen H., Sun H., You F., Sun W., Zhou X., Chen L., Yang J., Wang Y., Tang H., Guan Y. (2011). Activation of STAT6 by STING is critical for antiviral innate immunity. Cell.

[22] Yoneyama M., Suhara W., Fukuhara Y., Fukuda M., Nishida E., Fujita T. (1998). Direct triggering of the type I interferon system by virus infection: Activation of a transcription factor complex containing IRF-3 and CBP/p300. EMBO J..

[23] Taniguchi T., Takaoka A. (2002). The interferon-alpha/beta system in antiviral responses: A multimodal machinery of gene regulation by the IRF family of transcription factors. Curr. Opin. Immunol..

[24] Archambault V., Glover D.M. (2009). Polo-like kinases: Conservation and divergence in their functions and regulation. Nat. Rev. Mol. Cell Biol..

[25] Matsumoto G., Shimogori T., Hattori N., Nukina N. (2015). TBK1 controls autophagosomal engulfment of polyubiquitinated mitochondria through p62/SQSTM1 phosphorylation. Hum. Mol. Genet..

[26] Deng Z., Lim J., Wang Q., Purtell K., Wu S., Palomo G.M., Hu B., Tan S., Manfredi D., Zhao Y. (2019). ALS-FTLD-linked mutations of SQSTM1/p62 disrupt selective autophagy and NFE2L2/NRF2 anti-oxidative stress pathway. Autophagy.

[27] Goode A., Butler K., Long J., Cavey J., Scott D., Shaw B., Sollenberger J., Gell C., Johansen T., Searle M.S. (2016). Defective recognition of LC3B by mutant SQSTM1/p62 implicates impairment of autophagy as a pathogenic mechanism in ALS-FTLD. Autophagy.

[28] Marchlik E., Thakker P., Carlson T., Jiang Z., Ryan M., Marusic S., Goutagny N., Kuang W., Askew G.R., Roberts V. (2010). Mice lacking Tbk1 activity exhibit immune cell infiltrates in multiple tissues and increased susceptibility to LPS-induced lethality. J. Leukoc. Biol..

[29] Xia Y., Yan L., Huang B., Liu M., Liu X., Huang C. (2014). Pathogenic mutation of UBQLN2 impairs its interaction with UBXD8 and disrupts endoplasmic reticulum-associated protein degradation. J. Neurochem..

[30] Ravenhill B.J., Boyle K.B., Von Muhlinen N., Ellison C.J., Masson G.R., Otten E., Foeglein A., Williams R., Randow F. (2019). The Cargo Receptor NDP52 Initiates Selective Autophagy by Recruiting the ULK Complex to Cytosol-Invading Bacteria. Mol. Cell.

[31] Ahmad L., Zhang S.-Y., Casanova J.-L., Sancho-Shimizu V. (2016). Human TBK1: A Gatekeeper of Neuroinflammation. Trends Mol. Med..

[32] Trinchieri G. (2010). Type I interferon: Friend or foe?. J. Exp. Med..

[33] Yu J., Zhou X., Chang M., Nakaya M., Chang J.-H., Xiao Y., Lindsey J.W., Dorta-Estremera S., Cao W., Zal A. (2015). Regulation of T-cell activation and migration by the kinase TBK1 during neuroinflammation. Nat. Commun..

[34] Kurlawala Z., Shah P.P., Shah C., Beverly L.J. (2017). The STI and UBA Domains of UBQLN1 Are Critical Determinants of Substrate Interaction and Proteostasis. J. Cell. Biochem..

[35] Honda K., Takaoka A., Taniguchi T. (2006). Type I interferon [corrected] gene induction by the interferon regulatory factor family of transcription factors. Immunity.

[36] Gao L., Tu H., Shi S.T., Lee K.-J., Asanaka M., Hwang S.B., Lai M.M.C. (2003). Interaction with a ubiquitin-like protein enhances the ubiquitination and degradation of hepatitis C virus RNA-dependent RNA polymerase. J. Virol..

[37] Hjerpe R., Bett J.S., Keuss M.J., Solovyova A., McWilliams T.G., Johnson C., Sahu I., Varghese J., Wood N.T., Wightman M. (2016). UBQLN2 Mediates Autophagy-Independent Protein Aggregate Clearance by the Proteasome. Cell.

[38] Rothenberg C., Srinivasan D., Mah L., Kaushik S., Peterhoff C.M., Ugolino J., Fang S., Cuervo A.M., Nixon R.A., Monteiro M.J. (2010). Ubiquilin functions in autophagy and is degraded by chaperone-mediated autophagy. Hum. Mol. Genet..

[39] Wu S., Mikhailov A., Kallo-Hosein H., Hara K., Yonezawa K., Avruch J. (2002). Characterization of ubiquilin 1, an mTOR-interacting protein. Biochim. Biophys. Acta.

[40] Chang L., Monteiro M.J. (2015). Defective Proteasome Delivery of Polyubiquitinated Proteins by Ubiquilin-2 Proteins Containing ALS Mutations. PLoS ONE.

[41] Fecto F., Yan J., Vemula S.P., Liu E., Yang Y., Chen W., Zheng J.G., Shi Y., Siddique N., Arrat H. (2011). SQSTM1 mutations in familial and sporadic amyotrophic lateral sclerosis. Arch. Neurol..

[42] Maruyama H., Morino H., Ito H., Izumi Y., Kato H., Watanabe Y., Kinoshita Y., Kamada M., Nodera H., Suzuki H. (2010). Mutations of optineurin in amyotrophic lateral sclerosis. Nature.

[43] Heo J.-M., Ordureau A., Paulo J.A., Rinehart J., Harper J.W. (2015). The PINK1-PARKIN Mitochondrial Ubiquitylation Pathway Drives a Program of OPTN/NDP52 Recruitment and TBK1 Activation to Promote Mitophagy. Mol. Cell.

[44] Ryzhakov G., Randow F. (2007). SINTBAD, a novel component of innate antiviral immunity, shares a TBK1-binding domain with NAP1 and TANK. EMBO J..

[45] Morton S., Hesson L., Peggie M., Cohen P. (2008). Enhanced binding of TBK1 by an optineurin mutant that causes a familial form of primary open angle glaucoma. FEBS Lett..

[46] Ma X., Helgason E., Phung Q.T., Quan C.L., Iyer R.S., Lee M.W., Bowman K.K., Starovasnik M.A., Dueber E.C. (2012). Molecular basis of Tank-binding kinase 1 activation by transautophosphorylation. Proc. Natl. Acad. Sci. USA.

[47] Huang B., Wu Q., Zhou H., Huang C., Xia X.G. (2016). Increased Ubqln2 expression causes neuron death in transgenic rats. J. Neurochem..

[48] Markovinovic A., Ljutic T., Beland L.C., Munitic I. (2018). Optineurin Insufficiency Disbalances Proinflammatory and Anti-inflammatory Factors by Reducing Microglial IFN-beta Responses. Neuroscience.

[49] Wang R., Yang B., Zhang D. (2011). Activation of interferon signaling pathways in spinal cord astrocytes from an ALS mouse model. Glia.

[50] Gerbino V., Kaunga E., Ye J., Canzio D., O’Keeffe S., Rudnick N.D., Guarnieri P., Lutz C.M., Maniatis T. (2020). The Loss of TBK1 Kinase Activity in Motor Neurons or in All Cell Types Differentially Impacts ALS Disease Progression in SOD1 Mice. Neuron.

[51] De Majo M., Topp S., Smith B.N., Nishimura A., Chen H.-J., Gkazi S.A., Miller J., Wong C.H., Vance C., Baas F. (2018). ALS-associated missense and nonsense TBK1 mutations can both cause loss of kinase function. Neurobiol. Aging.

[52] Duan W., Guo M., Yi L., Zhang J., Bi Y., Liu Y., Li Y., Li Z., Ma Y., Zhang G. (2019). Deletion of Tbk1 disrupts autophagy and reproduces behavioral and locomotor symptoms of FTD-ALS in mice. Aging.

