# Supplementary material for: UBQLN2 Promotes the Production of Type I Interferon via the TBK1-IRF3 Pathway
Source: Cells. 2020 May 13;9(5):1205. doi: 10.3390/cells9051205 (PMC7290724; doi:10.3390/cells9051205)
Supplement: Supplementary file 1 [file cells-09-01205-s001.pdf]

UBQLN2 promotes the production of type I interferon via TBK1-IRF3 pathway

(Supplementary Figures)

Tianhong Chen[1, 2], Wenjuan Zhang[1, 2], Bo Huang[1], Xuan Chen[1], Cao Huang[1]

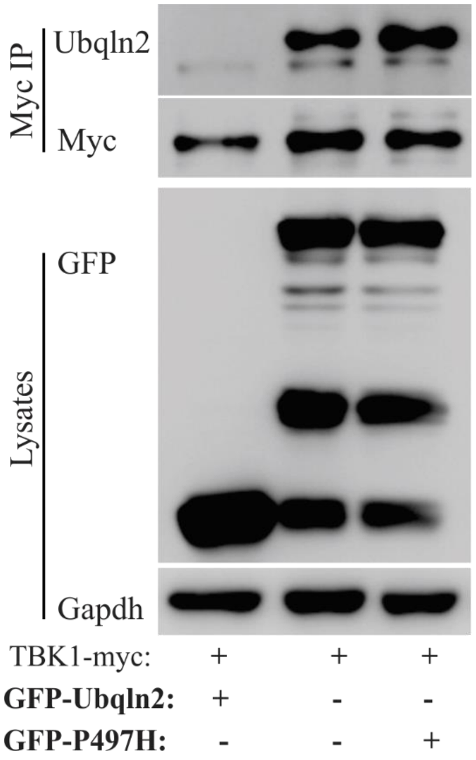

Fig. S1: TBK1 precipitates UBQLN2. Immunoprecipitation with anti-c-myc resin revealed that Ubqln2 was precipitated with TBK1 in HEK-293T cells when co-transfected myc-tagged TBK1 with the N-terminal GFP tagged Ubqln2: GFP-Ubqln2 (wild-type) or GFP-P497H (mutant). The transfected cells were harvested 48 hours after transfection.

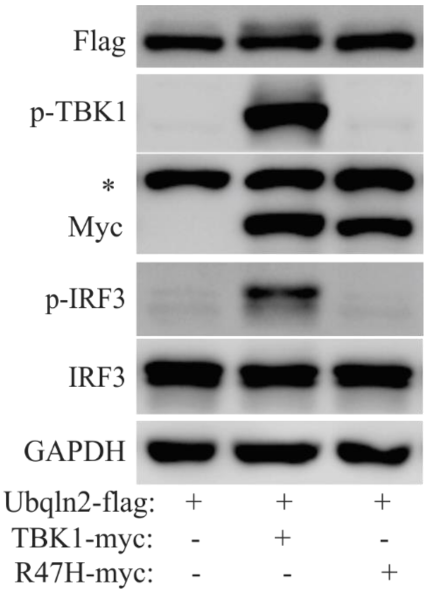

Fig. S2: UBQLN2 has no effect on mutant TBK1. Immunoblot analysis revealed the expression levels of TBK1 and IRF3 when co-expressed with wild-type or mutant (R47H) TBK1 and UBQLN2 in HEK-293T cells. The transfected cells were harvested 48 hours after transfection. Each lane was loaded with 10 µg total protein, and GAPDH was used as the loading control.

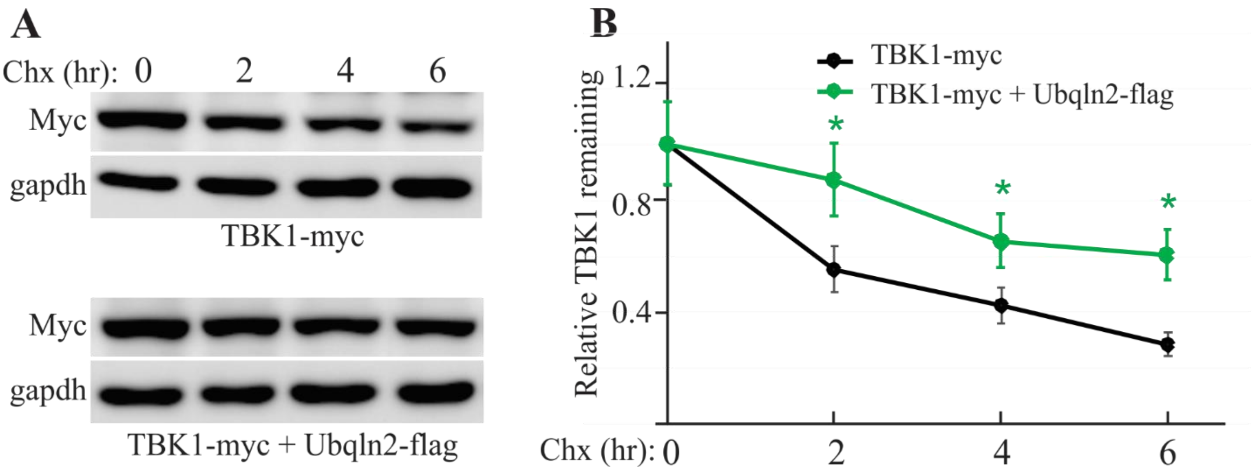

Fig. S3: UBQLN2 increases TBK1 protein stability. a. HEK-293T cells were co-transfected with TBK1-myc and either UBQLN2-3xFlag expression vector or empty vector. Cycloheximide (CHX, 100 µg/ml) was added 24 hours after transfection, and cells were harvested at the indicated times. Equal amounts of the protein lysates were analyzed for myc and for GAPDH. **b.** A graph showing the relative turnover of TBK1 protein after cycloheximide treatment. The data are reported as mean ± standard deviation (n = 3). $^*p < 0.05$.

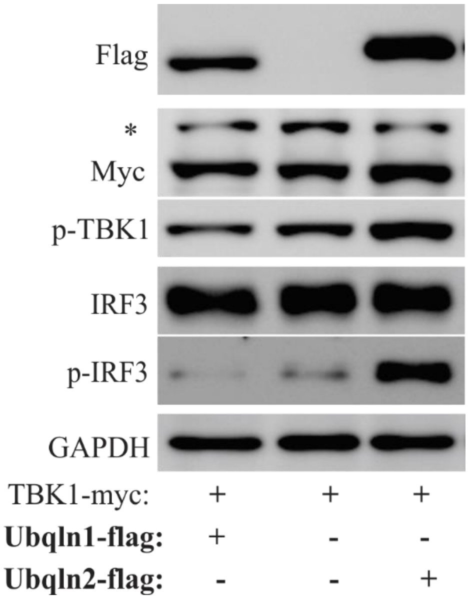

Fig. S4: Increased expression of Ubiquilin 1 does not affect TBK1 phosphorylation. Immunoblot analysis revealed the expression levels of TBK1 and IRF3 when co-expressed with TBK1 and Ubiquilin 1 (Ubqln1) or Ubqln2 in HEK-293T cells. Both human Ubqln1 and Ubqln2 were tagged with 3x Flag at the C-terminus and co-transfected with TBK1 into HEK-293T cells. The transfected cells were harvested 48 hours after transfection. Each lane was loaded with 10 µg total protein, and GAPDH was used as the loading control.

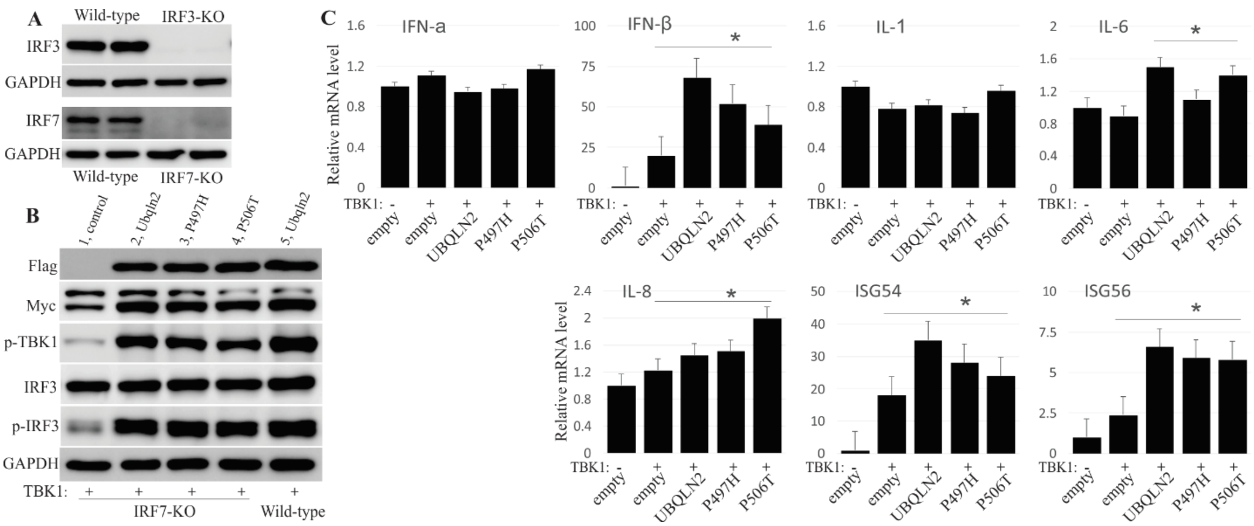

Fig. S5: Loss of IRF7 has no effect on IFN1 production enhanced by UBQLN2. a. CRISPR-Cas9–mediated IRF3 knockout (IRF3-KO) and IRF7 knockout (IRF7-KO) HEK-293T cells. **b.** Immunoblot analysis revealed the expression levels of TBK1 and IRF3 when co-expressed with UBQLN2. Cells were transfected with Myc-tagged TBK1 and Flag-tagged wild-type or mutant UBQLN2. Harvested cells 48 hours after transfection. **c.** Quantitative PCR analysis revealed the relative levels of mRNA for IFN1 and related cytokines in IRF7-KO cells. Harvested cells 24 hours after transfection. The data are reported as the mean ± standard deviation (n =3). *P < 0.05.
